# Data on the removal of peroxides from functionalized polyethylene glycol (PEG) and effects on the stability and sensitivity of resulting PEGylated conjugates

**DOI:** 10.1016/j.dib.2020.106258

**Published:** 2020-09-02

**Authors:** Samuel Babity, Davide Brambilla

**Affiliations:** Faculté de Pharmacie, Université de Montréal, 2940 Chemin de Polytechnique, Montréal, Canada

**Keywords:** PEGylation, ROS, Fluorescence, Monitoring, Reduction/Oxidation

## Abstract

Cyanine-5 (Cy5) is a fluorescent dye active in the far-red region of the visible spectrum (λ_ex_ = 646 nm, λ_em_ = 662 nm) [1]. While Cy5 displays fluorescence in its oxidized form, it can be readily converted to its non-fluorescent hydrocyanine equivalent (H-Cy5) when exposed to reducing agents. H-Cy5 can then be converted back to its fluorescent oxidized form when exposed to reactive oxygen species (ROS), allowing it to act as a highly sensitive, high wavelength fluorescent ROS sensor [2]. However, H-Cy5 is a small, poorly water-soluble molecule, and is rapidly taken up into cells *in vivo*, preventing its use for sensing extracellular ROS, which are implicated in inflammation, wound healing, and other processes [3], [4], [5], [6]. A solution to this lies in the conjugation of Cy5 to a polyethylene glycol (PEG) polymer, increasing its solubility [7]. This conjugate (Cy5-PEG) can be reduced to H-Cy5-PEG to allow the highly sensitive detection of ROS in an aqueous extracellular environment. However, after PEG conjugation, a significant decrease in stability and sensitivity is observed, likely owing to the presence of ROS contaminants in commercial samples of PEG. It has been reported that these ROS impurities can be removed from PEG through a simple freeze-drying procedure [8]. Here, we demonstrate that a simple, straightforward method for the purification of PEG can allow the synthesis of a highly functional, water-soluble ROS sensor that could be used for extracellular ROS sensing.

## **Specifications Table**

**Table:** 

Subject	Chemistry (General)
Specific subject area	Preparation of Polymer Conjugates for Pharmaceutical Applications
Type of data	Table Graph
How data were acquired	Fluorescence Spectroscopy Spark® multimode fluorescence plate reader; SparkControl software (Tecan Group, Ltd., Männedorf, Switzerland)
Data format	Raw Analyzed
Parameters for data collection	Fluorescence intensities (λ_ex_ = 630 nm, λ_em_ = 675 nm, gain 125) were measured at 23°C in a black 96-well plate (Brand GMBH & Co., Wertheim, Germany) using a Spark® multimode fluorescence microplate reader (Tecan Group Ltd., Männedorf, Switzerland).
Description of data collection	PEGylated conjugates were prepared, with or without purified PEG, and the stability and sensitivity data for these conjugates was collected by fluorescence spectroscopy.
Data source location	Institution: Université de Montréal City/Town/Region: Montreal Country: Canada
Data accessibility	Repository name: Mendeley Data Data identification number: 10.17632/fsg3r9j6py.1 Direct URL to data: https://data.mendeley.com/datasets/fsg3r9j6py/1

## Value of the Data

• These data are useful because the possibility of auto-oxidation is an important consideration when PEGylating sensitive compounds. In addition, they provide a protocol to generate a highly sensitive, easily tuneable, and water soluble ROS sensor;
• Analytical chemists, biochemists, and cell biology scientists preparing sensitive PEGylated conjugates of dyes, biosensors, or pharmaceuticals can benefit from these data, by potentially being able to increase the stability of their compounds;
• The data can be used to gain further insights because the purification of PEG could allow easier preparation of oxidation-sensitive PEGylated conjugates for subsequent studies. By following a simple protocol, scientists can easily design highly sensitive, water soluble, and easy-to-conjugate ROS sensors;

## 1 Data Description

**Fig. 1:**
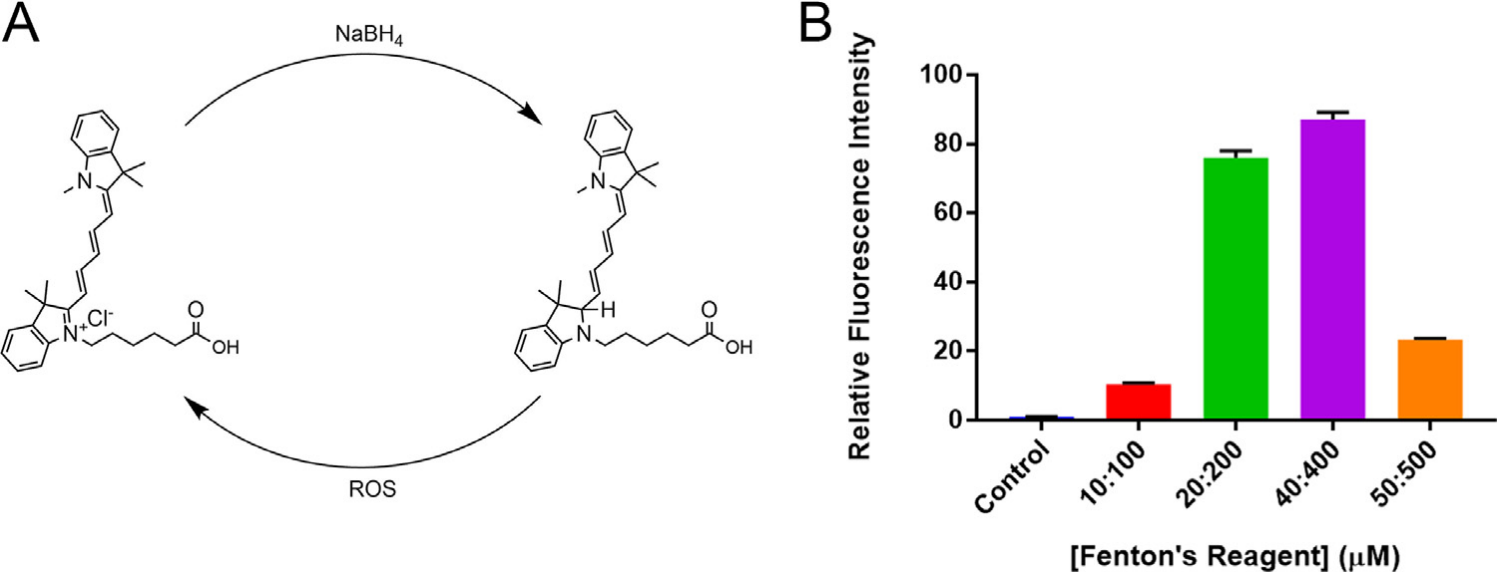
A) The reduction and oxidation cycle of Cy5 and H-Cy5; B) The relative sensitivity of free H-Cy5 in solution (10 µM). Mean ± SD (*n* = 3).

Cyanine-5 (Cy5) can be readily converted to hydrocyanine-5 (H-Cy5) by reaction with sodium borohydride (NaBH_4_); H-Cy5 can then be returned to the fluorescent oxidized Cy5 through exposure to ROS (including hydroxyl and superoxide radicals) (Fig. 1A) [2]. When incubated in an aqueous solution of Fenton's Reagent (a source of hydroxyl radicals), H-Cy5 displays up to 80-fold greater fluorescence than when incubated in water alone (Fig. 1B). Data were plotted using GraphPad Prism 7 (San Diego, CA).

**Fig. 2:**
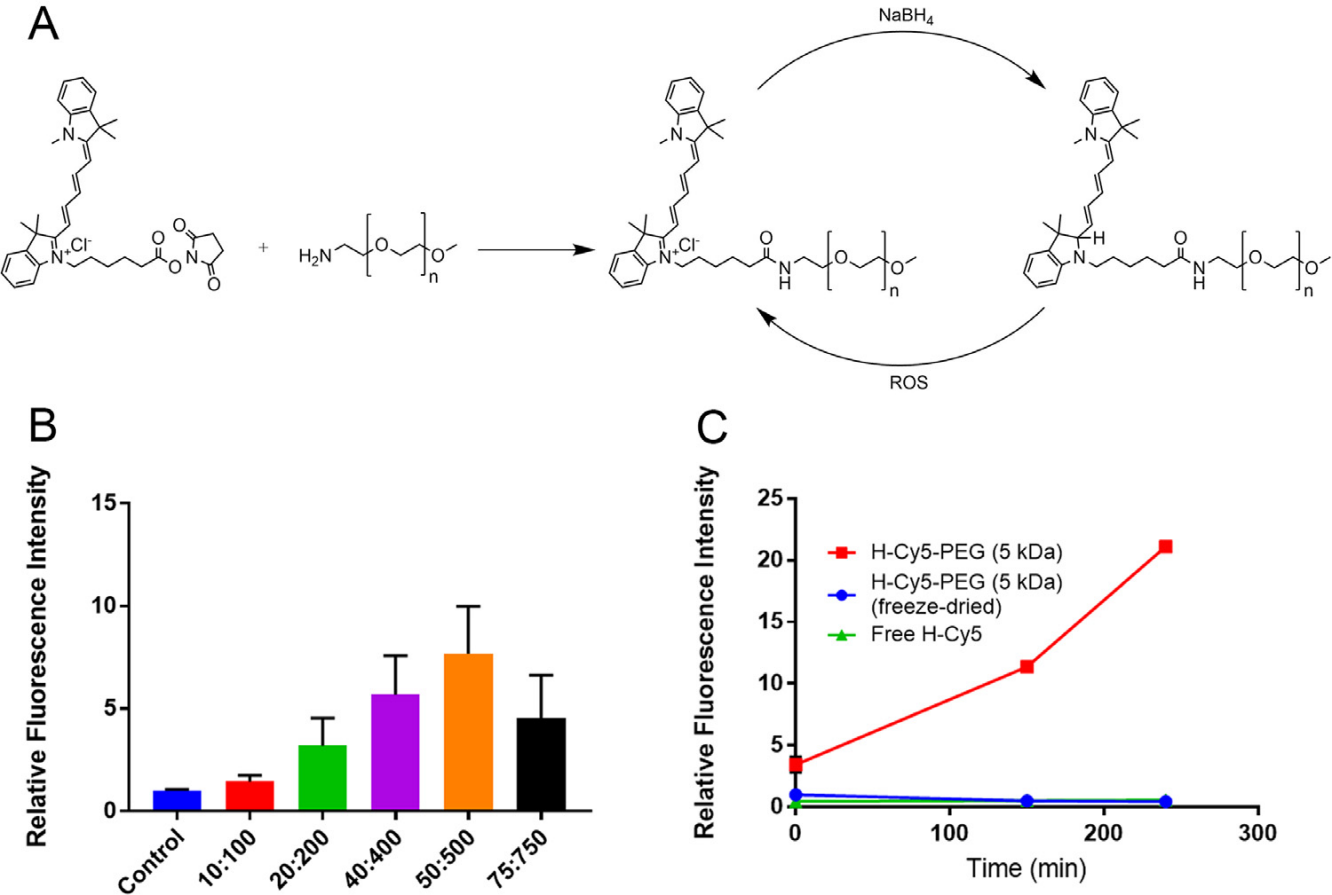
A) PEGylation reaction of Cy5, and reduction-oxidation cycle of Cy5-PEG and H-Cy5-PEG; B) The relative sensitivity of H-Cy5-PEG in solution (10 µM). Mean ± SD (*n* = 3); C) The relative stability of H-Cy5-PEG made using commercial or purified PEG, as compared to free H-Cy5 (10 µM). Mean ± SD (*n* = 3).

An activated NHS ester of Cy5 can be coupled to an amine-functionalized polyethylene glycol (PEG) polymer, resulting in a water-soluble conjugate Cy5-PEG [7]. Using NaBH_4_, this conjugate can be converted to the hydrocyanine equivalent (H-Cy5-PEG) in the same manner as free Cy5. H-Cy5-PEG can then be converted back to the fluorescent oxidized Cy5-PEG when exposed to ROS (Fig. 2A). When incubated in an aqueous solution of Fenton's Reagent, the relative sensitivity of H-Cy5-PEG (8-fold) was greatly reduced compared to free H-Cy5 (Fig. 2B). When the stability of H-Cy5-PEG was assessed in aqueous solution under ambient conditions, it was found that H-Cy5-PEG experienced considerable oxidation (20-fold) within 4 hours, while free H-Cy5 did not experience any significant oxidation. When H-Cy5-PEG was prepared using purified PEG, it displayed no observable difference in stability from free H-Cy5 (Fig. 2C).

**Fig. 3:**
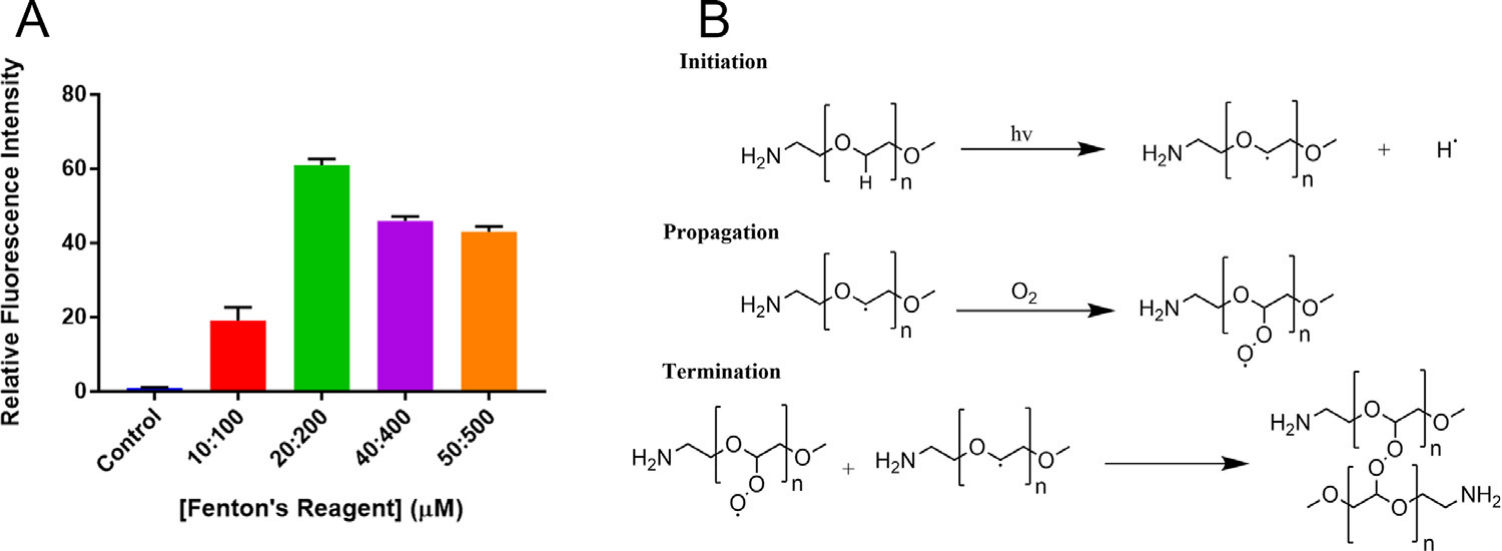
A) The relative sensitivity of H-Cy5-PEG made with purified PEG in solution (10 µM). Mean ± SD (*n* = 3); B) The proposed mechanism of ROS contamination in commercial PEG samples, and their conversion into inactive products by chain-termination reaction.

When the sensitivity of H-Cy5-PEG made from purified PEG was assessed by incubation in a solution of Fenton's reagent, it was found that H-Cy5-PEG displayed up to a 60-fold greater fluorescence intensity than when incubated in water alone (Fig. 3A). The generation of ROS in commercial PEG samples is proposed to proceed by a UV light-catalysed radical initiation reaction, followed by an oxygen-mediated propagation step, forming peroxide radicals [8]. The purification has been proposed to proceed through the acceleration of chain-terminating reactions under reduced pressure, resulting in the formation of inactive products (Fig. 3B).

## 2 Experimental design, materials, and methods

### 2.1 Materials

Dimethyl Sulfoxide (DMSO) was purchased from Sigma-Aldrich (St. Louis, MO). Hydrogen peroxide (30% v/v), sodium chloride (NaCl), and methanol (MeOH) were purchased from Fisher Scientific (Waltham, MA). Sodium borohydride (NaBH_4_) and iron sulfate heptahydrate (FeSO_4_•7H_2_O) were purchased from Acros Organics (Fair Lawn, NJ). Sephadex G-15 was purchased from GE Life Sciences (Pittsburgh, PA). Poly(ethylene glycol) amine (PEG-NH_2_) (5 kDa) was purchased from JenKem Technologies (Plano, TX). LC-MS analyses were performed on a 6120 Quadrupole LC/MS provided by Agilent Technologies (Santa Clara, CA). Lyophilization was conducted using a VirTis BenchTop Pro freeze-dryer purchased from SP Scientific (Warminster, PA).

## 3 Methods

### 3.1 Purification of PEG-NH_2_

Purification of PEG-NH_2_ was adapted from a previously described procedure [8]. Briefly, MeO-PEG-NH_2_ (Mw = 5000 g/mol) (60 mg, 12 µmol) was dissolved in 1 mL of milli-Q H_2_O and cooled to −80 °C. This solution was lyophilized at 40 mT for 48 h, yielding a fluffy white solid, which was stored at −20 °C under nitrogen.

### 3.2 Synthesis of Cy5-PEG

Cy5-PEG was synthesized by adapting a previously described procedure [7]. Briefly, Cy5-NHS (7.4 mg, 12 μmol, 2 eq) and MeO-PEG-NH_2_ (MW = 5000 g/mol) (30 mg, 6 μmol, 1 eq) were dissolved in 1 mL of anhydrous DMSO, and the mixture was stirred for 18 h in the dark. Then, the mixture was diluted in 20 mL of milli-Q H_2_O and lyophilized. The resulting residue was dissolved in 1 mL of 180 mM NaCl and purified on G-15 Sephadex. The resulting fractions were combined and lyophilized, giving Cy5-PEG as a blue solid (28 mg, 5.6 μmol, 86% yield). The purity of the product was confirmed by HPLC-MS analysis.

### 3.3 Reduction of Cy5 and Cy5-PEG

Cy5 and Cy5-PEG were converted to their reduced forms (H-Cy5 and H-Cy5-PEG) using a previously described procedure [3]. Unconjugated Cy5 (1 mg, 1.93 μmol, 1 eq) was dissolved in 300 μL of MeOH and 500 μL of a 1 mg/mL solution of NaBH_4_ in MeOH (13.2 μmol, 6.8 eq) was added. Cy5-PEG (5.3 mg, 0.96 μmol, 1 eq) was dissolved in 150 μL of 1:1 MeOH/degassed H_2_O, and 250 μL of a 1 mg/mL solution of NaBH_4_ in MeOH (7.9 μmol, 8 eq) was added. The loss of blue colour indicated that the reaction had occurred, yielding H-Cy5 or H-Cy5-PEG. The fluorescence intensity was measured using a Spark® multimode fluorescence microplate reader (Tecan Group, Ltd., Männedorf, Switzerland), and the reduction was considered complete if the value obtained was < 500 (λ_ex_ = 630 nm, λ_em_ = 675 nm, gain 125).

### 3.4 Re-oxidation of H-Cy5 and H-Cy5-PEG

The re-oxidation of H-Cy5 and H-Cy5-PEG was carried out by adapting a previously described procedure [3]. A 10 μM solution of H-Cy5 or H-Cy5-PEG was prepared in H_2_O. FeSO_4_ and H_2_O_2_ were added, yielding final concentrations of 10–75 μM and 100–750 µM respectively. The fluorescence (λ_ex_ = 630 nm, λ_em_ = 675 nm, gain 125) intensity was monitored at 23 °C in a black 96-well plate (Brand GMBH & Co., Wertheim, Germany) using a multimode fluorescence microplate reader.

### 3.5 Stability of H-Cy5 and H-Cy5-PEG

A 10 μM solution of H-Cy5 or H-Cy5-PEG was prepared in H_2_O. The solution was kept in the dark, and fluorescence (λ_ex_ = 630 nm, λ_em_ = 675 nm, gain 125) intensity was periodically monitored at 23°C in a black 96-well plate (Brand GMBH & Co., Wertheim, Germany) using a multimode fluorescence microplate reader.

## Declaration of Competing Interest

The authors declare that they have no known competing financial interests or personal relationships which have, or could be perceived to have, influenced the work reported in this article.

## Appendix Supplementary materials

Image, application 1

## References

[1] Kvach M.V., Ustinov A.V., Stepanova I.A., Malakhov A.D., Skorobogatyi M.V., Shmanai V.V., Korshun V.A. (2008). A convenient synthesis of cyanine dyes: reagents for the labelling of biomolecules. Eur. J. Org. Chem..

[2] Kundu K., Knight S.F., Willett N., Lee S., Taylor W.R., Murthy N. (2009). Hydrocyanines: a class of fluorescent sensors that can image reactive oxygen species in cell culture, tissue, and in vivo. Angew. Chem. Int. Ed. Engl..

[3] Andina D., Brambilla D., Munzinger N., Frei J., Zivko C., Leroux J.-C., Luciani P. (2017). Development of a modular ratiometric fluorescent probe for the detection of extracellular superoxide. Eur. J. Chem..

[4] Ekstrand M., Gustafsson Trajkovska M., Perman-Sundelin J., Fogelstrand P., Adiels M., Johansson M., Mattsson-Hulten L., Boren J., Levin M. (2015). Imaging of intracellular and extracellular ROS levels in atherosclerotic mouse aortas ex vivo: effects of lipid lowering by diet or atorvastatin. PLoS One.

[5] Duval C., Cantero A.-V., Auge N., Mabile L., Thiers J.-C., Negre-Salvayre A., Salvayre R. (2003). Proliferation and wound healing of vascular cells trigger the generation of extracellular reactive oxygen species and LDL oxidation. Free Radic. Biol. Med..

[6] Bauer G. (2014). Targeting extracellular ROS signaling of tumor cells. Anticancer Res..

[7] Proulx S.T., Luciani P., Christiansen A., Karaman S., Blum K.S., Rinderknecht M., Leroux J.-C., Detmar M. (2013). Use of a PEG-conjugated bright near-infrared dye for functional imaging of rerouting of tumor lymphatic drainage after sentinel lymph node metastasis. Biomaterials.

[8] Kumar V., Kalonia D.S. (2006). Removal of peroxides in polyethylene glycols by vacuum drying: implications in the stability of biotech and pharmaceutical formulations. AAPS PharmSciTech.

